# Comparison of the Bruker MALDI-TOF Mass Spectrometry System and Conventional Phenotypic Methods for Identification of Gram-Positive Rods

**DOI:** 10.1371/journal.pone.0106303

**Published:** 2014-09-03

**Authors:** Claudia Barberis, Marisa Almuzara, Olivier Join-Lambert, María Soledad Ramírez, Angela Famiglietti, Carlos Vay

**Affiliations:** 1 Instituto de Fisiopatología y Bioquímica Clínica, Hospital de Clínicas José de San Martín, Facultad de Farmacia y Bioquímica, Universidad de Buenos Aires, Ciudad Autónoma de Buenos Aires, Argentina; 2 Université Paris Descartes, Paris, France; 3 Laboratoire de Microbiologie, Hopital Necker-Enfants malades, Paris, France; 4 Instituto de Microbiología y Parasitología Médica, Universidad de Buenos Aires-Consejo Nacional de Investigaciones Científicas y Tecnológicas, Ciudad Autónoma de Buenos Aires, Argentina; Universitätsklinikum Hamburg-Eppendorf, Germany

## Abstract

In recent years, MALDI-TOF Mass Spectrometry (MS) method has emerged as a promising and a reliable tool for bacteria identification. In this study we compared Bruker MALDI-TOF MS and conventional phenotypic methods to identify a collection of 333 Gram-positive clinical isolates comprising 22 genera and 60 species. 16S rRNA sequencing was the reference molecular technique, and *rpo*B gene sequecing was used as a secondary gene target when 16Sr RNA did not allow species identification of *Corynebacterium* spp. We also investigate if score cut-offs values of ≥1,5 and ≥1,7 were accurate for genus and species-level identification using the Bruker system. Identification at species level was obtained for 92,49% of Gram-positive rods by MALDI-TOF MS compared to 85,89% by phenotypic method. Our data validates the score ≥1,5 for genus level and ≥1,7 for species-level identification in a large and diverse collection of Gram-positive rods. The present study has proved the accuracy of MALDI-TOF MS as an identification method in Gram-positive rods compared to currently used methods in routine laboratories.

## Introduction

*Corynebacterium* spp. and other Gram-positive rods (GPR) are widespread throughout nature, being most of the species part of the normal skin flora of humans and animals [1]. However, they are increasingly recognized as causes of human infection since immunosuppressive treatments, oncological diseases, antibiotic treatments, and multiple invasive procedures make patients vulnerable to opportunistic infections [2]. Correct identification of *Corynebacterium* spp. and other GPR species is a very challenging task because it will help to identify the real source of infection and install the appropriate treatment for the infection.

In routine laboratories the most used techniques to identify microorganisms are the conventional phenotypic tests [1], [3]. However, these tests are time consuming and do not always give reliable identification at the species level.

The use of the rRNA gene sequence for GPR identification is one of the most commonly used molecular technique and is considered the gold standard approach for its identification in many cases [4]. Sequencing of the 16S rRNA gene discerns among most species in the genus *Corynebacterium* [5]. In order to arrive to a correct molecular identification, since the 16S rRNA gene of corynebacteria has very little polymorphism, sequencing the complete 16S rRNA gene (approximately 1,500 bp) is required [6]. The sequencing of a fragment of the *rpo*B gene resulted to be an alternative method for the identification of corynebacteria. This later method should be used to resolve ambiguous cases for definitive identification [6]. However, it has some limitation, as it is expensive for routine laboratories.

In the last years MALDI-TOF Mass Spectrometry (MS) has emerged as a promising technique for bacterial identification. MALDI-TOF MS is a rapid, reliable diagnostic tool for the identification of most microorganisms [7]. The technology is unique in clinical microbiology, allowing laboratories to definitively identify bacterial isolates within minutes. The rapid turn-around time and minimal cost for consumables per specimen compared with conventional identification methods have resulted in MALDI-TOF MS being increasingly used in clinical laboratories worldwide [8].

Using the Bruker Biotyper MALDITOF MS system, some organisms such as Gram-negative bacteria, are easily analyzed by directly smearing a colony onto the MS target [9] but other types of bacteria such as GPR generally require preparatory tube extraction or a direct on plate testing using formic acid [10].

Previous studies reported that the Bruker MALDI-TOF MS system accurately identified GPR species such as *Corynebacterium* spp. [7], [11], [12]. However, information of GPR other than *Corynebacterium* spp. is scarcely documented or poor identification results were obtained in the available studies [13].

In a recent study, Alatoom et al. [11] evaluated the MALDI-TOF MS method using Bruker Biotyper system (Bruker Daltonics, Billerica, MA) for the identification of 92 clinical isolates of *Corynebacterium* species and compared with the identification using 16S rRNA gene sequencing. The authors suggested a new lower score for genus and species identification level, being the proposed scores ≥ 1,7 and ≥1,5, respectively.

The aims of the present study were to compare the performance of MALDI-TOF MS and conventional methods for the identification of GRP and also validate the previous proposed score cut-off of ≥ 1,7 for species level identification in a greater collection of heterogeneous group of GPR. All the results were compared with molecular techniques as the gold standard.

## Materials and Methods

### Bacterial isolates

A total of 333 GPR clinical strains from a culture collection obtained during the period 2009–2013 at the University teaching hospital, Hospital de Clínicas “José de San Martín”, Buenos Aires, Argentina, were used in this study. All isolates were recovered during clinical practice. We included every GPR species that were considered clinically significant as they were either recovered as unique pathogen from normally sterile body sites (e.g., blood culture or urines samples with a bacterial count of >10^4^/ml) or from adequately collected clinical material where they are the predominant organisms.

Briefly, bacterial strains were grown on 5% sheep blood agar in 5% CO_2_ at 35°C for 24 h (or later for slower growing species). The strains were stored at −70°C in brain heart infusion broth containing 20% glycerol until use.

All isolates were identified by conventional phenotypic methods as described before [1], [3]. Phenotypic identification of colonies included morphology, Gram staining, catalase activity, lipophily for *Corynebacterium* spp. and biochemical methods using the algorithm previously described by Funke et al [3], [14]. In parallel with conventional biochemical methods, molecular identification was performed.

Molecular identification was used as the gold standard method to compare the results obtained by MALDI TOF MS and conventional phenotypic methods. 16S rRNA gene sequencing was carried out for characterization of all isolates. In *Corynebacterium* spp. identification, *rpo*B gene was used as a secondary gene target when 16S rRNA gene did not allow correct identification to species level. PCR reactions were performed as previously described [15], [16]. Sequencing of the PCR products were performed on both DNA strands using ABIPrism 3100 BioAnalyzer equipment at Macrogen Inc. sequencing facility, South Korea, sequencing facility. The sequences were analyzed using the BLAST v2.0 software (http://www.ncbi.nlm.nih.gov/BLAST/). A ≥99,0% (16S rRNA gene) and ≥95,0% (*rpo*B gene) similarity cut-off was required for species identification.

### MALDI-TOF Mass Spectrometry

All isolates were retrospectively identified by MALDI-TOF MS. The clinical strains from the culture collection were subcultured on Columbia agar containing 5% sheep blood (Laboratorios Britania, Argentina) at 37°C with 5% CO_2_ for 24 to 48 h for MALDI-TOF MS measurement. Bacterial isolates were identified by the direct colony on plate extraction method as previously described [10]. MALDI-TOF target plates were inoculated into the spots by picking a freshly grown overnight colony and overlaid with 1 µl of 70% formic acid (Sigma-Aldrich). Each spot was allowed to dry and subsequently overlaid with 1 µl of matrix (α-cyano-4-hydroxycinnamic acid).

Mass spectra were acquired using the MALDI-TOF MS spectrometer in a linear positive mode (Microflex, Bruker Daltonics). The bacterial test standard (BTS, Bruker) was used for instrument calibration. Mass spectra were analyzed in a m/z range of 2,000 to 20,000. The MALDI Biotyper library version 3.0 and MALDI Biotyper software version 3.1 were used for bacterial identification. Based on previous studies [11] cut-off scores for identification were: ≥1,5 for genus level, ≥1,7 for species-level. A score <1,5 was considered as resulting in no reliable identification. A minimum difference of 10% between the top and next closest score was required for a different genus or species [11].

### Data analysis

Statistical analysis to calculate the efficiency of MALDI TOF MS and conventional phenotypic methods was carried out only in those genera comprising more than ≥ 20 isolates. Confidence intervals for identification were calculated with DAG Stat spreadsheet [17].

## Results

Among the 333 clinical GPR included in our study, we identified 22 genera and 60 species, most of which are known to cause human infections. This shows the great diversity of genus and species that we have analyzed.

For all the isolates the rate of identification at the species level was higher for MALDI-TOF MS compared to conventional phenotypic method (92,49% vs 85,89%). All the obtained results were compared with the 16S RNA/*rpo*B sequence analysis as the gold standard.

Among 216 *Corynebacterium* isolates tested, identification by MALDI-TOF MS at species level was 93,52% (202/216) whereas identification by conventional methods was 92,13 (199/216) (Tables 1, 2).

**Table 1:** Identification of 333 Gram- positive rods using MALDI-TOF MS.

Organism (No. of isolates tested)	No. of isolates by level of identification			
	Species level	Genus level	No ID	Error
	(≥1,7)	(≥1,5)	(<1,5)	
*Corynebacterium* spp.				
*C. striatum* (61)	61	61	0	0
*C. amycolatum* (38)	37	38	0	1^a^
*C. urealyticum* (14)	13	13	1	0
*C. pseudodiphtheriticum* (13)	12	13	0	1^b^
*C. glucuronolyticum* (12)	12	12	0	0
*C. jeikeium* (12)	12	12	0	0
*C. tuberculostearicum* (9)	7	7	2	0
*C. aurimucosum* (8)	4	8	0	0
*C. propinquum* (8)	8	8	0	0
*C. accolens* (7)	7	7	0	0
*C. afermentans* subsp *lipophilum* (4)	2	3	1	1^c^
*C.* group F (4)	4	4	0	0
*C. mucifaciens* (4)	4	4	0	0
*C. diphtheriae* (3)	3	3	0	0
*C. simulans* (3)	3	3	0	0
*C. afermentans* subsp *afermentans* (2)	1	1	1	0
*C. coyleae* (2)	1	2	0	1^d^
*C. imitans* (2)	2	2	0	0
*C. kroppenstedtii* (2)	2	2	0	0
*C. bovis* (1)	1	1	0	0
*C. durum* (1)	1	1	0	0
*C. macginleyi* (1)	1	1	0	0
*C. minutissimum* (1)	0	1	0	1^e^
*C. pseudotuberculosis* (1)	1	1	0	0
*C. riegelli* (1)	1	1	0	0
*C. ulcerans* (1)	1	1	0	0
*C. xerosis* (1)	1	1	0	0
Total (n: 216)	202 (93,52)	211(97,68)	5(2,3)	5(2,3)
*Actinomyces turicensis* (7)	6	7	0	1^f^
*Actinomyces radingae* (11)	9	10	1	1^g^
*Actinomyces urogenitalis* (6)	6	6	0	0
*Actinomyces odontolyticus* (5)	5	5	0	0
*Actinomyces europaeus* (3)	2	2	1	0
*Actinomyces naeslundii/viscosus* (5)	5	5	0	0
*Actinomyces neuii* (10)	10	10	0	0
*Actinobaculum schaalii* (10)	9	9	1	0
*Actinomyces gravenitzii* (1)	1	1	0	0
*Varibaculum cambriense* (1)	1	1	0	0
*Arcanobacterium haemolyticum* (6)	6	6	0	0
*Trueperella bernardiae* (2)	2	2	0	0
*Bifidobacterium scardovii* (1)	1	1	0	0
*Propionibacterium acnes* (1)	1	1	0	0
*Propionibacterium avidum* (2)	2	2	0	0
Total (n:71)	66(92,96)	68(95,77)	3(4,2)	2(2,8)
*Leifsonia* spp. (1)	0	0	1	0
*Exiguobacterium aurantiacum*. (2)	2	2	0	0
*Exiguobacterium* spp. (1)	0	0	1	0
*Brevibacterium casei* (2)	2	2	0	0
*Brevibacterium ravenspurgense* (1)	1	1	0	0
*Microbacterium aerolatum* (1)	1	1	0	0
*Microbacterium aurum* (1)	1	1	0	0
*Microbacterium testaceum* (1)	1	1	0	0
*Microbacterium oxydans* (1)	1	1	0	0
*Microbacterium hominis* (1)	0	1	0	0
*Microbacterium* spp. (1)	0	1	0	0
*Arthrobacter protophormiae*. (1)	1	1	0	0
*Cellulosimicrobium cellulans* (2)	2	2	0	0
*Turicella otitidis* (2)	2	2	0	0
*Rothia aeria* (2)	2	2	0	0
*Dermabacter hominis* (10)	10	10	0	0
*Clostridium tertium* (1)	1	1	0	0
*Listeria monocytogenes* (4)	4	4	0	0
*Lactobacillus gasseri* (1)	1	1	0	0
*Lactobacillus rhamnosus* (1)	1	1	0	0
*Lactobacillus paracasei* (1)	1	1	0	0
*Rhodococcus equi* (2)	2	2	0	0
*Gordonia terrae* (2)	2	2	0	0
*Gordonia* spp. (1)	0	1	0	0
*Dietzia maris* (1)	1	1	0	0
*Dietzia natronolimnaea* (1)	1	1	0	0
*Dietzia* spp. (1)	0	0	1	0
Total (n:46)	40(86,9)	43(93,5)	3(6,5)	0
TOTAL (n:333)	308 (92,49)	322 (96,69)	11(3,3)	7(2,1)

a Corynebabacterium aurimucosum; **^b^**Corynebacterium propinquum; ^c^Corynebacterium jeikeium; ^d^Corynebacterium afermentans, ^e^Corynebacterium amycolatum,^ f^ Actinomyces radingae, ^g^Actinomyces europaeus.

**Table 2:** Tabla 2. Comparative identification rates of Gram-positive rods by conventional phenotypic method and MALDI-TOF MS.

Organism	No.	No.(%) ID conventional method		No.(%) ID MALDI-TOF method	
		Species	Genus/Group*	Species	Genus
*Corynebacterium* spp.	216	199 (92,13)	216 (100)	202 (93,52)	211 (97,68)
*Actinomyces* spp. and related genera	71	63 (88,73)	69 (97,18)	66 (92,96)	68 (95,78)
**Other Gram positive rods**					
*Dermabacter hominis, Rothia aeria, Turicella otitidis*	14	14 (100)	14 (100)	14 (100)	14 (100)
Pigmented Gram-positive rods *(Leifsonia* spp., *Microbacterium* spp., *Exiguobacterium* spp., *Cellulosimicrobium* spp., *Brevibacterium/Arthrobacter* spp.*)*	16	0 (0)	16 (100)*	12 (75)	14 (87,5)
Aerobic actinomycetes (*Gordonia* spp., *Rhodococcus equi, Dietzia* spp.)	8	2 (25)	6 (75)*	6 (75)	7 (87,5)
*Listeria monocytogenes, Lactobacillus* spp.,*Clostridium tertium*	8	8 (100)	8 (100)	8 (100)	8 (100)
Total	333	286 (85,89)		308 (92,49)	

* Correct identification was considered as a group.

Validating the proposed MALDI-TOF MS identification score for *Corynebacterium* at genus and species level [11], 153/202 isolates (75,7%) yielded a score ≥2,0, all of which were correctly identified to the species level. Forty-nine isolates (24,3%) yielded a score between <2,0-≥1,7. There were 4 isolates that could not be identified at species level because the difference between the top and the next score gave two different results (*Corynebacterium aurimucosum/Corynebacterium. minutissimum*) with less than 10%. Also, five isolates were misidentified (Table 3).

**Table 3:** % of Gram-positive rods identified to the species level according to cut-off values by MALDI-TOF MS.

Organism	No.(%) with cut-off scores ≥2,0 (species level)	No.(%) with cut-off scores <2,0-≥1,7 (species level)	No. (%) with cut-off scores ≥1,7-≥2,0 (species level)
*Corynebacterium* spp.	153 (75,7)	49 (24,3)	202 (100)
*Actinomyces* spp. and related genera	41 (63,1)	24 (36,9)	65 (100)
**Other Gram positive rods**			
*Dermabacter hominis*	10	0	10
*Rothia aeria*	2	0	2
*Turicella otitidis*	2	0	2
*Microbacterium* spp.	1	3	4
*Exiguobacterium* spp.	1	1	2
*Brevibacterium/Arthrobacter* spp.	4	0	4
*Cellulosimicrobium* spp.	2	0	2
*Gordonia* spp.	1	1	2
*Rhodococcus equi*	2	0	2
*Dietzia* spp.	0	2	2
*Listeria monocytogenes*	4	0	4
*Lactobacillus* spp.	2	1	3
*Clostridium tertium*	1	0	1
Total Other GPR	32 (80)	8 (20)	40 (100)

For *Actinomyces* and related genera (*Actinobaculum* spp., *Varibaculum* spp., *Arcanobacterium* spp., *Trueperella* spp., *Bifidobacterium* spp.), the results of MALDI-TOF MS at genus level was 95,77% (68/71) and to species level 92,96% (66/71). In these genera the phenotypic identification was less reliable to allow the correct species level identification (Table 1, 2). The MALDI-TOF MS identification scores were ≥2,0 in 41/65 isolates (63,1%), all of which were correctly identified to the species level. Twenty-four isolates (36,9%) yielded a score between <2,0-≥1,7 (Table 3).

For other GPR, identification by MALDI-TOF MS at genus level was 93,5% (43/46) and 86,9% (40/46) to species level. Thirty-two isolates (80%) generated identification scores >2,0 and 8 isolates (20%) scores of <2,0- ≥1,7 (Table 3). In the particular case of pigmented Gram-positive rods and aerobic actinomycetes when we use the 16S RNA amplification to arrive to the correct identification, we observed that in some isolates such as *Leifsonia* spp., *Exiguobacterium* spp., *Dietzia* spp., *Gordonia* spp., we did not yield accurate results at species level (Table 1).

For the bacterial isolates in which we obtained the species level, we apply the DAG stat spreadsheet tool to visualize the efficiency of the MALDI-TOF MS and the conventional method compared with the molecular methods (Table 4).

**Table 4:** Efficiency of MALDI-TOF MS and conventional method for isolates of *Corynebacterium* spp., and *Actinomyces* spp. and related genera.

Organism	No.	Efficiency (95% CI) conventional-molecular method	Efficiency (95% CI) MALDI-TOF- molecular method
*Corynebacterium* spp.	216	0,92 (CI: 0,88–0,95)	0,94 (CI:0,89–0,96)
*Actinomyces* spp. and related genera	71	0,89 (CI:0,79–0,95)	0,93 (CI:0,84–0,98)

## Discussion

MALDI-TOF MS technology is now recognized as an efficient method for bacterial identification in routine laboratory [13].

Several authors have analyzed the differences between pre-analytical procedure, direct colony with and without formic acid and tube extraction method [11]. Direct on-plate testing and tube extraction of *Corynebacterium* spp. have yielded equivalent identification percentages at genus and species level using Andromas system [10]. Farfour et al. showed that the MALDI-TOF MS Andromas strategy was reliable to identify a set of 659 Gram-positive rods representing 16 bacterial genera and 72 species by the direct colony method [18]. In that report, Andromas MALDI-TOF MS system could not identify *Listeria* isolates to the species level because of very similar mass spectra [18]. In this study 4/4 *Listeria monocytogenes* isolates could be identified to the species level using Bruker Biotyper system. However, as no other *Listeria* species isolates were included in our collection of GPR, the real accuracy of this technique to identify *Listeria* remains to be established.

Species identification rates of *Corynebacterium* spp. isolates were similar by MALDI-TOF MS and conventional phenotypic methods. On the other hand in *Actinomyces* and related genera the efficiency was higher using MALDI-TOF MS (Table 4). However, MALDI-TOF MS is easy to perform, rapid and less costly than conventional phenotypic method for routine laboratories. These features make this method a better option for bacterial identification.

Moreover, not only in pigmented GPR (*Exiguobacterium, Microbacterium, Leifsonia*, *Cellulosimicrobium*), but also in aerobic actinomycetes (*Dietzia, Gordonia*) the identification at species level was higher using the MALDI-TOF MS method. In these isolates the conventional phenotypic methods are difficult to perform because there are many similarity biochemical tests to correctly achieve the identification at species or genus level.

Finally, based on previous studies, a lower score cut-off is suitable for identification of GPR and equivalent in test accuracy than manufacturer recommended cut-off [11]. As the genus and species level identification cut-off score was reduced, correct identification increased more than 20% for all the studied isolates (Table 3). Our data suggests and expands upon results from Alatoom et al., since a great and diverse collection and other Gram-positive rods have been tested [11]. Other authors also showed that using the manufactureŕs cut-off, they obtained a lower level of identification at the species level. However, genus level identification remained equivalent [10].

In our experience the reduction in identification score required for species level and the direct on plate testing using formic acid, allows to identify the most commonly GPR isolated in the clinical microbiology laboratory today.

To conclude, this study demonstrates the accuracy of Bruker MALDI-TOF MS system as an identification method for clinical GPR using direct colony extraction method. Our study clearly expose that the MALDI-TOF MS database has a wide range of different spectrum allowing the identification of most of the clinical genera and species recovered in the clinical settings. However, since new pathogens are emerging continuously, revision and addition of new spectra to the database must be considered.
